# Hypoechogenicity of brainstem raphe correlates with depression in migraine patients

**DOI:** 10.1186/s10194-019-1011-2

**Published:** 2019-05-15

**Authors:** Wei-Wei Tao, Xin-Ting Cai, Jie Shen, Xue-Gong Shi, Yu Wang

**Affiliations:** 1 Department of Neurology, Epilepsy and Headache Group, The First Affiliated Hospital of Anhui Medical University, Jixi Road 218, Hefei, 230022 China; 2 Department of Echocardiography, The First Affiliated Hospital of Anhui Medical University, Jixi Road 218, Hefei, 230022 China; 3 Department of Neurology, The Fourth Affiliated Hospital of Anhui Medical University, Huaihai Avenue 100, Hefei, 230000 China

**Keywords:** Brainstem raphe, Depression, Hypoechogenicity, Hyperechogenicity, Migraine without aura, Transcranial sonography

## Abstract

**Background:**

Brainstem raphe (BR) hypoechogenicity in transcranial sonography (TCS) has been depicted in patients with major depression (MD) and in depressed patients with different neurodegenerative diseases. But, up to date, the association of BR alterations in TCS with depression in migraineurs has never been reported. This study was to investigate the possible role of BR examination via TCS in migraineurs with depression.

**Methods:**

Forty two migraine without aura (MwoA) patients and 40 healthy controls were recruited. Echogenicity of lentiform nuclei (LN), caudate nuclei (CN), substantia nigra (SN) and brainstem raphe (BR) and width of the frontal horns of the lateral ventricles and the third ventricle were assessed with TCS. The diagnosis of depression was based on the criteria of the Diagnostic and Statistical Manual of Mental Disorders IV (DSM –IV), and the severity of depression was measured by Hamilton Rating Scale for Depression (HAM-D) and Hospital Anxiety and Depression Scale depression subscale (HADS-D).

**Results:**

There were no significant differences between migraineurs and controls in the width of frontal horn of the lateral ventricle (*p* = 0.955), width of third ventricle (*p* = 0.129) as well as in the echogenicity of SN (*p* = 0.942), CN (*p* = 0.053), LN (*p* = 0.052) and BR (*p* = 0.677). Here, it seems that more migraineurs were detected with increased echogenecity of CN and LN compared with controls (33.3% versus 15.0% for CN, 19.0% versus 5.0% for LN) though they had no statistical significance. Patients with hypoechogenic BR had significantly higher HAM-D and HADS-D scores than those with normal BR signal (*p* = 0.000 for both HAM-D and HADS-D), and most (83.33%) migraineurs with depression exhibited hypoechogenic raphe but none (0.00%) of the migraineurs without depression exhibited hypoechogenic raphe (*p* = 0.000).

**Conlusions:**

TCS signal alteration of BR can be a biomarker for depression in migraine but it is not associated with migraine headache itself. LN and CN alterations in TCS may reflect a potential role of them in the pathogenesis of migraine, which needs to be further elucidated.

## Background

Transcranial sonography (TCS) is a reliable and non-invasive neuroimaging technique for detecting small deep brain parenchyma such as basal ganglia (BG), substantia nigra (SN) and brainstem raphe (BR) [1]. The hyperechogenicity of SN has high accuracy in the diagnosis of PD [2]. The hypoechogenicity of BR has been found to be associated with depression in a number of neurological disorders such as Parkinson’s disease (PD) [3], Huntington’s disease [4], idiopathic Rapid Eye Movement (REM) sleep behavior disorder [5], myotonic dystrophies [6] and cerebral small vessel disease [7]. Recently, the hypoechogenicity of BR has been proposed as biomarker for depression in PD [3], and even it can be used to improve detection of depressive symptoms in early PD stages where affective disturbances may not be recognized by clinicians in the context of PD phenomena [8].

The comorbid relationship between migraine and depressive disorder are well-known. The risk of comorbid depressive disorder in migraineurs is over 2.5 times higher than in non-migraineurs [9, 10], vise versa, the risk of comorbid migraine in major depressive disorder (MDD) patients is 2 to 3 times higher than in non-MDD controls [11]. Depression has been shown to be the most relevant psychiatric comorbidities associated with migraine, influencing the prevelance, treatment and prognosis of migraine [12]. On the other hand, growing evidences indicate that comorbid depression is a risk factor for chronification of migraine and development of medication overuse headache [13]. But, up to date, it has never been reported about the association of BR alterations in TCS with depression in migraineurs, though BR hypoechogenicity has been shown to be correlated to higher headache attack frequency in migraineurs without depression in one study [14] but not in another one study [15].

In order to investigate the possible role of BR in migraine with depression, migraine without aura (MwoA) patients with or without depression will be included in this study. Possible alterations of BR echogenicity in TCS in migraineurs will be studied in comparison with the healthy control. And possible difference in BR echogenicity between migraineurs with and without depression will be studied. On the other hand, possible alterations of the width of frontal horn of the lateral ventricle and the third ventricle as well as of the echogenicity of substantia nigra(SN), caudate nuclei (CN) and lentiform nuclei (LN) will also be studied, as alterations of these deep brain structures have been identified either in migraine [16–19] or in depression [20, 21].

## Methods

### Subjects

A total of 42 patients fulfilling the International Headache Society (IHS) classification criteria for MwoA [22] were prospectively screened from the Outpatient Clinic of the Department of Neurology, the First Affiliated Hospital of Anhui Medical University, China. Migraine with aura (MA) patients were not included in this study in order to avoid possible statistical analysis confusion caused by fewer cases of MA as much fewer MA patients are available in clinic. All included patients had recurrent headaches and none of them had ever accepted regular prophylactic therapy before this study. All migraineurs had no history of other types of headaches. Magnetic resonance imaging (MRI) was performed in a headache-free period for all patients. With the exception of depression, all other comorbid conditions that may lead to brain structure alterations were excluded, such as diabetes mellitus, hypertension, high LDL-cholesterol, hyperuricemia, kidney disease, hepatopathy, autoimmune disease, cardiac source of embolism, smoking and obesity. The demographic and clinical data of the patients were the following: female to male 26: 16; mean age 34.1 ± 9.1, range 24–55 years; disease duration 7.9 ± 7.7, range 0.5–25 years; attack frequency/month 1.0, range 1.0–3.0 (Table 1). As controls, 40 age and gender-matched healthy subjects were included (mean age 31.6 ± 8.6, range 22–55 years (Table 1). All control subjects had no history of recurrent headaches. Subjects with inadequate temporal bone window were excluded. Depression was measured by using the Hamilton Rating Scale for Depression (HAM-D) and the depression subscale of the Hospital Anxiety and Depression Scale depression subscale (HADS-D). The diagnosis of depression was based on the criteria of the Diagnostic and Statistical Manual of Mental Disorders IV (DSM –IV).

**Table 1:** Demographic and clinical characteristics of migraine patients and controls

	Migraine (*N* = 42)	Control (*N* = 40)	*P* value
Gender (F:M)	26:16	20:20	0.278^#^
Age, year	34.1 ± 9.1 (24–55)	31.6 ± 8.6 (22–55)	0.222^*^
Attack frequency	1.0 (1.0–3.0)	–	–
Disease duration	7.9 ± 7.7 (0.5–25)	–	–
HAM-D score	9.1 ± 7.0 (0–24)	4.9 ± 3.4 (0–11)	0.001^*^
HADS-D score	6.5 ± 4.5 (0–18)	3.2 ± 2.2 (0–7)	0.000^*^

Values are given as mean ± standard deviation (minimum-maximum). Attack frequency values are given as migraine headache attack times per month (median, IQR). # chi-square test, * t-test

### Transcranial sonography

TCS examination was conducted by Dr. Xue-Gong Shi, an experienced sonographer who has been engaged in ultrasound for 26 years and he was blinded to clinical scores of the enrolled participants in this study. A phased-array ultrasound system equipped with a 1.6–2.5 MHz transducer (EPIQ 7C, Philips, USA) was employed for the examination. A dynamic range of 45–55 dB and penetration depth of 14–16 cm through the transtemporal bone window were used [23, 24]. For each examination, the image brightness and time gain compensations were adapted as needed.

**Fig. 1:**
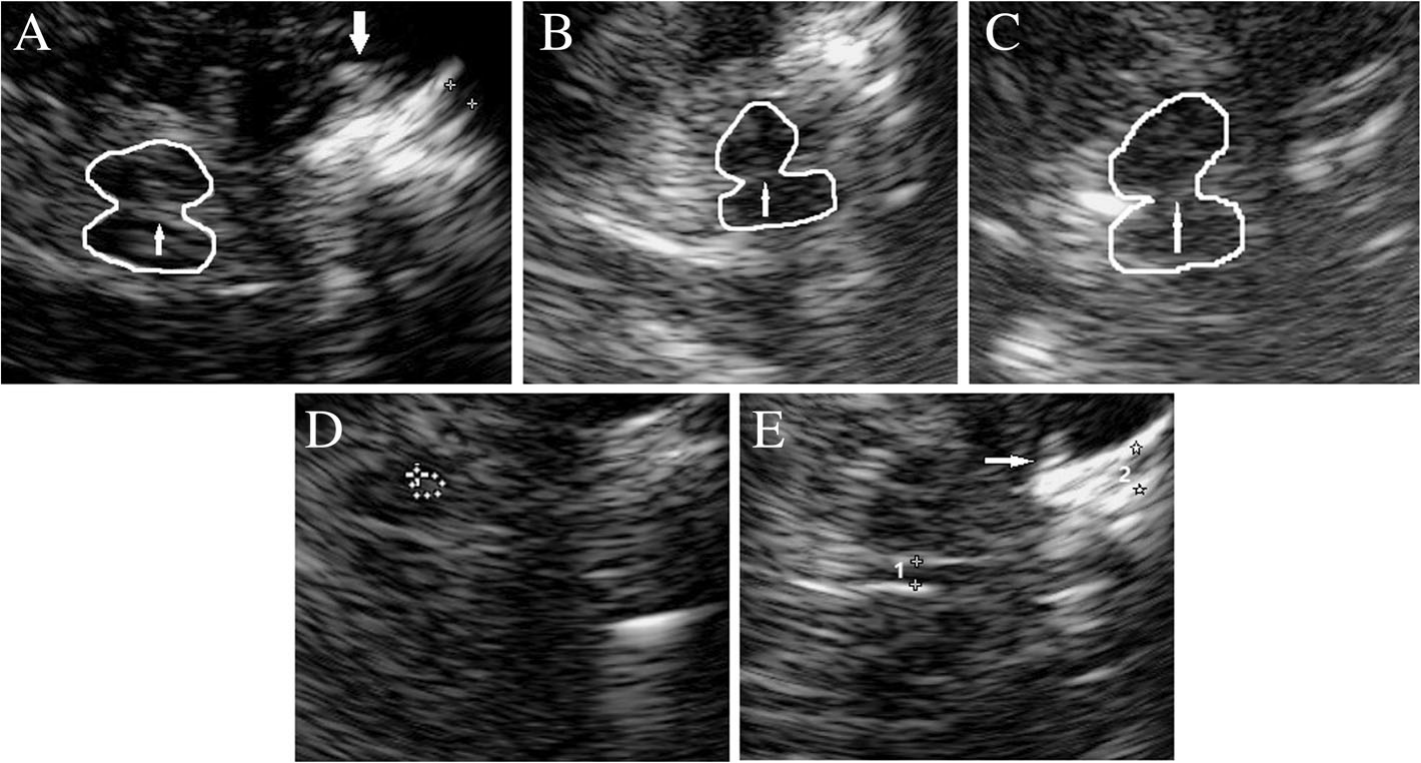
TCS images of mesencephalic (**a**, **b**, **c**, **d**) and diencephalic plane (**e**). **a** Normal echogenicity of BR (thin arrow), grade 2; thick arrow indicates hyperechogenicity of LN. **b** Pathologic echogenicity of BR, the echogenic line of the raphe appears abnormally slight and thin, grade 1. **c** Pathologic echogenicity of BR, the raphe is not visible, grade 0. **d** Normal echogenic area of SN. **e** Hyperechogenicity of CN (thin arrow), width of the frontal horn of the lateral ventricle (2) and of the third ventricle (1)

TCS examination was carried out on standardized axial imaging planes as recommended for deep brain parenchyma detection [24]. At the diencephalic plane, we measured the width of the frontal horns of the lateral ventricles, the transverse diameter of the third ventricle (Fig. 1e), and the echogenicity of LN and CN. Echogenic alterations of LN and CN were identified as hyperechogenic if they looked more intense than the surrounding white matter (Fig. 1a, e). Normal ranges of ventricle widths are age-dependent. In subjects under/over the age of 60, widths of > 7/> 10 mm (3rd ventricle) and > 17/> 20 mm (frontal horn) are regarded as abnormal [25]. At the mesencephalic plane, we measured the echogenecity of SN and BR. The size of SN smaller than 0.20 cm^2^ is considered normal, larger than 0.20 cm^2^ is rated as hyperechogenicity [26] (Fig. 1d). The echogenicity of the BR was assessed semiquantitatively using three-point scale: 0 = raphe not visible (Fig.1c), 1 = reduced or interrupted echogenicity (the echogenic line of the brainstem raphe is interrupted or appears abnormally slight, thin or dotted) (Fig. 1b) and 2 = normal echogenicity (continuous line with an echogenicity similar to that of red nucleus). The raphe echogenicity graded 0 or 1 was classified as abnormal, and graded 2 was normal [25] (Fig. 1a).

### Statistical analysis

The data is shown as mean ± standard deviation (SD) if it is normally distributed, or else as median (intequartile range, IQR). For comparison of categorical variables, the chi-square test was performed. For comparison of non-normally distributed data, the Mann-Whitney U test was used. And for comparison of means, the t-test for independent samples was used. A *p*-value of less than 0.05 was considered to have statistically significant difference.

## Results

### Depression

Significant difference was observed between migraineurs and controls in HAM-D score (mean 9.1, SD 7.0, range 0–24 in migraineurs; mean 4.9, SD 3.4, range 0–11 in controls; *p* = 0.001) and HADS-D score (mean 6.5, SD 4.5, range 0–18 in migraineurs; mean 3.2, SD 2.2, range 0–7 in controls; *p* = 0.000) (Table 1.). Based on DSM-IV criteria for depression and IHS criteria for MwoA [22], twelve patients were diagnosed as MwoA with depression and thirty as MwoA without depression.

### TCS findings

The width of frontal horn of the lateral ventricle was found normal (≤17 mm) in all migraineurs and controls, mean width was 4.62 ± 0.98 mm in migraineurs and 4.63 ± 1.30 mm in controls (*p* = 0.955). The width of the third ventricle (> 7 mm) was found abnormal in one migraine patients (4.8%) and normal in the rest migraineurs and controls. Mean width of third ventricle is 4.13 ± 1.26 mm and 3.77 ± 0.81 mm in migraineurs and controls respectively (*p* = 0.129). Hyperechogenic SN was present in 4 migraineurs (9.5%) and 4 controls (10.0%). Mean area of SN is 0.16 ± 0.03 cm^2^ in migraineurs and 0.15 ± 0.04 cm^2^ in controls (*p* = 0.308). CN hyperechogenicity was found in 14 migraineurs (33.3%) and 6 controls (15.0%). And hyperechogenicity of LN was found in 8 migraineurs (19.0%) and 2 controls (5.0%). Hypoechogenicity of BR was present in 10 patients (23.8%) and 8 controls (20.0%), and BR echogenicity was undetectable in 1 out of the 10 patients with BR hypoechogenicity but in none of the healthy controls. There were no significant difference between migraineurs and controls in the width of frontal horn of the lateral ventricle (*p* = 0.955), width of third ventricle (*p* = 0.129) as well as the echogenicity of SN (*p* = 0.942), CN (*p* = 0.053), LN (*p* = 0.052) and BR (*p* = 0.677) (Table 2).

**Table 2:** Comparison of TCS findings between migraineurs and controls

Brain structure	Migraine (*N* = 42)	Control group (*N* = 40)	*P* value
Width of frontal horn of LV (mm)	4.62 ± 0.98	4.63 ± 1.30	0.955^*^
Width of TV (mm)	4.13 ± 1.26	3.77 ± 0.81	0.129^*^
Area of SN (cm^2^)	0.16 ± 0.03	0.15 ± 0.04	0.308^*^
Normal	38	36	0.942^#^
Hyperechogenic	4	4	
Echogenicity of BR			0.677^#^
Normal	32	32	
Hypoechogenic	10	8	
Echogenicity of CN			0.053^#^
Normal	28	34	
Hyperechogenic	14	6	
Echogenicity of LN			0.052^#^
Normal	34	38	
Hyperechogenic	8	2	

Values are given as mean ± standard deviation or exact number. # chi-square test, * t-test. *LV* lateral ventricle, *TV* Third ventricle, *SN* Substantia Nigra, *BR* brain raphe, *CN* Caudate nucleus, *LN* Lentiform nucleus

### Correlation between depression and BR hypoechogenecity

Our study showed that patients with hypoechogenic BR had higher HAM-D and HADS-D scores than those with normal BR. Mean HAM-D score was 18.6 ± 4.6 (range 11–24)in patients with hypoechogenic BR, and 6.1 ± 4.6 (range 0–14)in patients with normal BR (*p* = 0.000). Mean HADS-D score was 12.6 ± 3.8 (range 7–18)in patients with hypoechogenic BR, and 4.6 ± 2.7 (range 0–11)in patients with normal BR (*p* = 0.000). Additionally, one patient with undetectable BR echogenicity had obviously depressive symptoms with HAM-D score at 22 and HADS-D score at 18. Nevertheless, we found no significant differences between two groups in gender, age, disease duration and migraine headache attack frequency (Table 3). Twelve migraine patients fulfilled the diagnostic criteria of depression based on DSM-IV. Ten of the twelve (83.33%) depressed migraineurs exhibited reduced echogenicity of BR, whereas none of the thirty (0.00%) migraineurs without depression showed hypoechogenicity of BR (*p* = 0.000).

**Table 3:** Comparison of clinical characteristics between migraineurs with and without hypoechogenicity of BR

	Normal BR (*N* = 32)	Hypoechogenic BR (*N* = 10)	*P* value
Gender (F:M)	22:10	4:6	0.102^#^
Age, year	33.5 ± 8.7 (24–50)	35.8 ± 10.7 (26–55)	0.494^*^
Attack frequency	1.0 (1.0–2.8)	2.0 (1.0–11.3)	0.212^^^
Disease duration	7.8 ± 8.0 (0.5–20)	8.5 ± 6.8 (6–20)	0.791^*^
HAM-D score	6.1 ± 4.6 (0–14)	18.6 ± 4.6 (11–24)	0.000^*^
HADS-D score	4.6 ± 2.7 (0–11)	12.6 ± 3.8 (7–18)	0.000^*^

Values are given as mean ± standard deviation (minimum-maximum). Attack frequency values are given as migraine headache attack times per month (median, IQR). # chi-square test, * t-test, ^ Mann-Whitney U test

## Discussion

To our knowledge, this is the first study to correlate alterations of BR echogenicity with depression in migraine patients. The main TCS finding is that migraineurs exhibiting a pathologic echogenicity of BR tend to have more depressive symptoms than those patients with normal raphe echogenicity. In addition, it could be shown that the overwhelming majority of migraineurs with depression exhibited hypoechogenic raphe, while none of the migraineurs without depression exhibited hypoechogenic raphe.

Some MRI studies have shown structural and functional alterations of BG in migraine patients [18, 27, 28], and this alterations may be indicators of migraine chronification [17].The globus pallidus is found to be associated with disease duration and the putamen associated with headache frequency in migraineurs, which further validated the probable role of the BG in the pathogenesis of migraine [18]. However, in our brain parenchyma sonography study, we found no significant changes in width of frontal horn of lateral ventricle, width of third ventricle, area of SN, and echogenecity of LN and CN. Here, it seems that more migraineurs were detected with increased echogenecity of CN and LN compared with controls (33.3% versus 15.0% for CN, 19.0% versus 5.0% for LN) though they had no statistical significance. One sonography study finds that CN and LN echogenicity and SN area are significantly altered in migraineurs [19]. LN and CN alterations in TCS may further support a potential role of them in pathogenesis of migraine, which needs to be further elucidated.

Multiple genetic and biochemical studies have demonstrated that serotonergic system is involved in migraine pathophysiology [29]. And many studies have indicated that the BR manipulation can regulate trigeminocervical complex (TCC) response to nociceptive inputs [30]. Further, selective degeneration of BR serotonergic neurons increases the propagating velocity of cortical spreading depression (CSD), a proposed basis of migraine aura [31]. These previous data implicate that BR might be involved in the pathogenesis of migraine. Hamerla et al. found that reduced BR echogenicity was more often in migraine patients than in healthy controls, and proposed that this finding may be a reflection of an alteration of the serotonergic raphe nuclei [15]. In another brain sonography study, the occurrence rate of BR hypoechogenicity did not differ between migraineurs and healthy subjects, but associated with higher headache attack frequency [14]. Whereas, neither the increased occurrence rate of BR hypoechogenicity in migraineurs compared with healthy control nor the association of BR hypoechogenicity with migraine headache attack frequency was found in current study. This inconsistency might be due to a narrow range of headache attack frequency in our study subjects. Thus, it can not be excluded that BR function is involved in migraine pain manipulation.

Amounts of studies have shown that raphe hypoechogenicity existed in MDD patients and in depressed patients with different neurodegenerative disease such as PD, Wilson’s disease, Dystonia and Huntington disease [32]. In our migraine patients, ten of the twelve depressed patients had hypoechogenic raphe, while none of the thirty non-depressed patients had hypoechogenic raphe. On the other hand, migraine patients with hypoechogenic raphe had higher depression score compared with migraine patients without hypoechogenic raphe, and one patient with undetectable BR echogenicity had obviously depressive symptoms with high score on HAM-D and HADS-D. This indicates a significant association of BR sonographic signal alterations with clinically evident depression in migraine patients. Therefore, TCS examination of brainstem might enable the identification of a subgroup of migraine patients who are at higher risk to suffer from or to develop depression.

### Limitations

We acknowledge that the migraine headache frequency was relatively low and its range was narrow in our studying subjects, which may affect the analysis of the association of migraine itself with the echogenecity of BR as well as with that of CN and LN. In fact, very subtle structural alterations with corresponding anatomical locations such as SN, CN, LN and BR can exist, which may be overlooked when examining at the sonographic level. The aim of this study was not to investigate the possible role of this deep brain parenchyma in the pathogenesis of migraine but to determine whether the alteration of BR echogenecity could be a biomarker of depression in migraine patients. It should also be acknowledged that no double control was utilized and only one sonographer conducted the ultrasound examination in this study.

## Conclusions

In TCS level, there was no significant difference between migraine patients and normal controls in deep brain parenchyma including brain ventricles and brainstem nuclei, but it seems that more migraineurs were detected with increased echogenecity of CN and LN compared with controls. Migraine patients with BR hypoechogenicity had significantly higher HAM-D and HADS-D scores than those with normal BR echogenicity, and overwhelming majority of migraineurs with depression exhibited hypoechogenic raphe but none of the migraineurs without depression exhibited hypoechogenic raphe. This indicates that TCS signal alteration of BR can be a biomarker for depression in migraine but not be associated with migraine headache itself. LN and CN alterations in TCS may reflect a potential role of them in the pathogenesis of migraine, which needs to be further elucidated.

## Abbreviations

BG: Basal ganglia
BR: Brainstem raphe
CN: Caudate nuclei
CSD: Cortical spreading depression
DSM-IV: Diagnostic and statistical manual of mental disorders IV
HADS-D: Hospital anxiety and depression scale depression subscale
HAM-D: Hamilton rating scale for depression
IHS: International headache society
LN: Lentiform nuclei
MA: Migraine with aura
MD: Major depression
MDD: Major depressive disorder
MRI: Magnetic resonance imaging
MwoA: Migraine without aura
REM: Rapid eye movement
SN: Substantia nigra
TCC: Trigeminocervical complex
TCS: Transcranial sonography
